# SUMOylation of RepoMan during late telophase regulates dephosphorylation of lamin A

**DOI:** 10.1242/jcs.247171

**Published:** 2021-09-09

**Authors:** Takanobu Moriuchi, Fumiko Hirose

**Affiliations:** Graduate School of Life Science, University of Hyogo, 3-2-1 Koto, Kamigori, Hyogo 678-1297, Japan

**Keywords:** Lamin, Mitosis, Nuclear lamina, Protein phosphatase, Sumoylation, SUMO-interacting motif, SIM

## Abstract

Dephosphorylation of lamin A, which triggers nuclear lamina reconstitution, is crucial for the completion of mitosis. However, the specific phosphatase and regulatory mechanism that allow timely lamin A dephosphorylation remain unclear. Here, we report that RepoMan (also known as CDCA2), a regulatory subunit of protein phosphatase 1γ (PP1γ) is transiently modified with SUMO-2 at K762 during late telophase. SUMOylation of RepoMan markedly enhanced its binding affinity with lamin A. Moreover, SUMOylated RepoMan contributes to lamin A recruitment to telophase chromosomes and dephosphorylation of the mitotic lamin A phosphorylation. Expression of a SUMO-2 mutant that has a defective interaction with the SUMO-interacting motif (SIM) resulted in failure of the lamin A and RepoMan association, along with abrogation of lamin A dephosphorylation and subsequent nuclear lamina formation. These findings strongly suggest that RepoMan recruits lamin A through SUMO–SIM interaction. Thus, transient SUMOylation of RepoMan plays an important role in the spatiotemporal regulation of lamin A dephosphorylation and the subsequent nuclear lamina formation at the end of mitosis.

## Abstract (precis)

**Summary:** Transient SUMOylation of RepoMan controls the recruitment of lamin A to telophase chromosomes, lamin A dephosphorylation and nuclear lamina formation.

## INTRODUCTION

Recent studies have highlighted the considerable roles of protein kinases and protein phosphatases in the entrance, proceedings and exit from mitosis (Gelens et al., 2018; Moura and Conde, 2019). Several serine/threonine kinase families, including cyclin-dependent kinases (Cdks), Polo kinases and Aurora kinases strictly control particular mitotic events by specifically phosphorylating key mitotic substrates (Combes et al., 2017; Gheghiani et al., 2017; Petronczki et al., 2008). Similarly, brief treatment of cells with okadaic acid, a specific inhibitor of the protein phosphatase 2A (PP2A) type phosphatase, disturbs orderly mitotic events involving mitotic spindle formation, chromosome architecture, chromosome separation and nuclear envelope reconstitution, indicating that the duration of the phosphorylation state of substrate proteins might be regulated by a variety of protein phosphatases as well as protein kinases (Foss et al., 2016; Ghosh et al., 1992; Moura and Conde, 2019; Vandre and Wills, 1992).

The nuclear lamina constitutes a fibrous structure located beneath the inner nuclear membrane that interacts with chromatin, the nuclear pore complex, and LAP2, emerin and MAN1 (LEM)-domain proteins and contributes both to nuclear structural stability and the regulation of chromatin organization in higher eukaryotes (Andrés and González, 2009; Wilson and Foisner, 2010; Yáñez-Cuna and van Steensel, 2017). The nuclear lamina is composed of A-type (lamin A and C, both encoded by *LMNA*) and B-type (lamin B1 and B2) lamins. Lamin polypeptides have well-defined conserved features including an N-terminal head, a central α-helical rod, and a C-terminal tail region containing an Ig-fold domain, with both types of lamins being self-assembled into nuclear lamina (Dechat et al., 2008; Turgay et al., 2017). However, during mammalian cell mitosis, the nuclear lamina is disassembled and then reassembled in a process that is regulated by the reversible phosphorylation of lamin proteins. Phosphorylation of lamin proteins at specific sites, shown to be mediated by mitotic kinases or Cdk1 both *in vivo* and *in vitro*, triggers nuclear envelope breakdown at the beginning of mitosis (Goss et al., 1994; Hocevar et al., 1993; Peter et al., 1990). In comparison, the dephosphorylation of lamin proteins, required for nuclear lamina reassembly during telophase and early G1, is considered to be mediated by protein phosphatase 1 (PP1) (Thompson et al., 1997). However, although B-type lamin has been reported to be dephosphorylated by the PP1–A-kinase-anchoring protein 149 (AKAP149, also known as AKAP1) complex (Steen et al., 2003), the location(s) at which A-type lamins are dephosphorylated during telophase along with the mechanism by which this process is regulated have yet to be elucidated.

The SUMO family of proteins covalently is attached to target proteins via specific lysine residues. In humans, three functional SUMO isoforms (SUMO-1, -2 and -3) are expressed ubiquitously. SUMO-2 and SUMO-3 share ∼92% identity but they share only 48% identity with SUMO-1. SUMO proteins are conjugated to the target proteins (SUMOylation) by a sequential cascade mediated by an E1 (the heterodimeric activating enzyme SAE1–SAE2), an E2 (the monomeric SUMO conjugating enzyme Ubc9, also known as UBE2I) and multiple E3 enzymes (Johnson and Blobel, 1997; Johnson et al., 1997). SUMOylation regulates numerous cellular processes, including DNA replication and repair, gene expression and response to stress. Steady-state levels of SUMOylation on most substrates are relatively low, owing to the existence of a variety of cysteine isopeptidases, known as SENPs, which remove SUMO proteins from substrates (Gong and Yeh, 2006; Li and Hochstrasser, 1999; Mikolajczyk et al., 2007). Therefore, it is considered that SUMOylation might be responsible for transient substrate changes including marked alteration in subcellular localization, stability, activity, protein–protein interaction and protein–chromatin interaction (Hay, 2005). In particular, non-covalent interactions occur between SUMOylated proteins and other proteins containing SUMO-interacting motifs (SIMs), which are characterized by the core consensus sequence (V/I-V/I-X-V/I) and generally flanked by acidic residues near the N- or/and C-terminus (Hecker et al., 2006; Song et al., 2004, 2005; Tang et al., 2008). Notably, these SUMO–SIM interactions have been shown to be involved in the formation of protein complexes in nuclei, such as the promyelocytic leukemia nuclear body (Lin et al., 2006), kinetochores (Schweiggert et al., 2016; Zhang et al., 2008) and transcription regulatory complexes (Kuo et al., 2005; Ouyang and Gill, 2009; Stielow et al., 2008; Yamashita et al., 2016).

In addition, recent studies have demonstrated the importance of spatio-temporally regulated SUMOylation of substrates along with its association with SIM-containing proteins for the proper progression of mitosis. In particular, detailed analyses of mitotic cells indicated that SUMO-1, and SUMO-2 and -3 (SUMO-2/3), are conjugated to distinct proteins during mitosis. For example, Borealin, a subunit of the chromosome passenger complex that ensures correct chromosome alignment and segregation, is reported to be SUMOylated during metaphase and anaphase (Klein et al., 2009); SUMO-2/3 conjugation to topoisomerase II allows its specific localization to the centromeres (Dawlaty et al., 2008), whereas SUMO-2/3-modified Nuf2 and BubR1 were found to recruit CENP-E to kinetochores (Zhang et al., 2008). Moreover, Lee et al. has reported that non-covalent binding between the anaphase promoting complex/cyclosome (APC/C) subunits APC4 and APC2 via SUMO–SIM interaction is critical for timely APC/C activation and anaphase progression (Lee et al., 2018).

Recently, we demonstrated that phosphorylated lamin A accumulates on telophase chromosomes via an interaction between a SIM located in the C-terminal Ig-fold domain (termed SIM3) of lamin A and unknown chromatin-binding protein(s) conjugated with SUMO-2 (Moriuchi et al., 2016). We also provided evidence indicating that this SUMO–SIM interaction is required for dephosphorylation of lamin A during telophase and subsequent nuclear lamina formation. Here, we show that RepoMan (also known as CDCA2), a specific regulatory subunit for PP1γ (Trinkle-Mulcahy et al., 2006), is transiently SUMOylated at telophase. Moreover, the SUMOylated RepoMan contributes to lamin A recruitment to telophase chromosomes and dephosphorylation of the mitotic lamin A phosphorylation. Our findings provide evidence that transient SUMOylation of RepoMan during late telophase is critical for the spatiotemporal dephosphorylation of lamin A and nuclear lamina formation at the end of mitosis.

## RESULTS

### RepoMan is SUMOylated at K762

To identify the SUMOylated factor(s) that recruit lamin A to telophase chromosomes via SUMO–SIM interaction as suggested in our previous study (Moriuchi et al., 2016), we searched recent reports concerning proteomics analyses of SUMOylated proteins along with the Database of Interacting Proteins (https://www.uniprot.org/database/DB-0016). We predicted that the RepoMan–PP1γ complex is the most probable candidate as: (1) it is required for normal progression of the late part of mitosis (Vagnarelli et al., 2011); (2) it accumulates on anaphase and telophase chromosomes (Qian et al., 2013; Vagnarelli et al., 2011), and (3) RepoMan is SUMOylated during M phase (Schimmel et al., 2014; Schou et al., 2014).

**Fig. 1.**
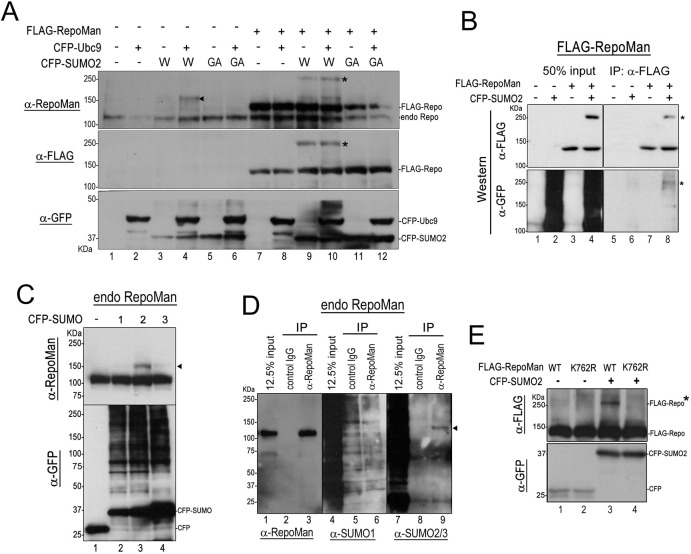
**SUMOylation of RepoMan at K762.** (A) HeLa cells were transfected with the indicated combinations of expression plasmids encoding CFP–SUMO-2 [WT (W) or GA mutant], CFP–Ubc9 and FLAG–RepoMan and directly lysed in boiled Laemmli's sample buffer. Polypeptides in whole-cell lysates were separated on a 5–20% gradient SDS polyacrylamide gel and subjected to western blot analysis using anti-RepoMan, anti-FLAG and anti-GFP antibodies. The asterisks and arrowhead indicate the CFP–SUMO-2-modified FLAG–RepoMan and endogenous RepoMan, respectively. (B) 293FT cells were transfected with plasmids expressing FLAG–RepoMan (WT) and CFP–SUMO-2 plasmid as indicated. At 24 h after DNA transfection, cell lysates were prepared and subjected to immunoprecipitation (IP) using an anti-FLAG antibody, then proteins were separated by SDS-PAGE and detected by western blots using anti-FLAG and anti-GFP antibodies. The asterisks indicate the CFP–SUMO-2-modified FLAG–RepoMan. (C) Conjugation of endogenous RepoMan with CFP–SUMO isoforms in HeLa cells were analyzed by western blot analysis. HeLa cells transfected with plasmids expressing CFP, CFP–SUMO-1, CFP–SUMO-2 or CFP–SUMO-3 were lysed in boiled Laemmli's sample buffer and proteins were separated on a 5–20% gradient gel by SDS-PAGE and subjected to western blot analysis using anti-RepoMan and anti-GFP antibodies. The arrowhead indicates the endogenous RepoMan conjugated to CFP-SUMO isoforms. (D) HeLa cells were lysed and subjected to immunoprecipitation using normal rabbit IgG or rabbit anti-RepoMan antibody, then proteins were separated on a 5–20% gradient gel by SDS-PAGE and detected by western blotting using anti-RepoMan, anti-SUMO-1 and anti-SUMO-2/3 antibodies. The arrowhead indicates the 125 kDa band specifically recognized by anti-SUMO-2/3. (E) 293FT cells were transfected with plasmids expressing FLAG–RepoMan(WT) or FLAG–RepoMan(K762R) with CFP or CFP–SUMO-2 and treated as in B. The asterisk indicates the FLAG–RepoMan modified with CFP-SUMO-2. Blots shown are representative of triplicate experiments.

Accordingly, we first confirmed SUMOylation of endogenous RepoMan by transiently expressing FLAG–RepoMan in HeLa cells transfected with or without a plasmid expressing wild type (WT) SUMO-2 or its conjugation-defective mutant (G93A) (Ihara et al., 2007) fused with CFP, and with or without a CFP–Ubc9 expression plasmid (Yamashita et al., 2016) (Fig. 1A). Immunoblot analysis using an anti-RepoMan antibody detected a major 150 kDa species in the lysate from HeLa cells simultaneously transfected with CFP–SUMO-2 (WT) and CFP–Ubc9 plasmids (lane 4), in addition to the 112 kDa endogenous RepoMan polypeptides. The apparent molecular mass of 150 kDa is equivalent to that of one RepoMan molecule and one CFP–SUMO-2 molecule, suggesting that RepoMan may be conjugated to a single molecule of SUMO-2. Signals of transiently expressed FLAG–RepoMan were similarly detected with anti-RepoMan and anti-FLAG antibodies (lanes 7–12). Both antibodies detected a signal with an apparent molecular mass of 250 kDa in addition to the 125 kDa signal of SUMOylation-free FLAG–RepoMan in lysates from CFP–SUMO-2(WT)-expressing cells (lanes 9 and 10). The 250 kDa band was absent in lysates from CFP- (lanes 7 and 8) or CFP–SUMO-2(G93A)-expressing cells (lanes 11 and 12), suggesting that this band is SUMOylated FLAG–RepoMan. To confirm that the slower migrating bands were SUMOylated FLAG–RepoMan, immunoprecipitation experiments using 293FT cells expressing FLAG–RepoMan and CFP–SUMO-2 were performed. As shown in Fig. 1B, western blotting with anti-GFP antibody allowed for the detection of slower migrating band in the precipitate with anti-FLAG antibody only when FLAG–RepoMan and CFP–SUMO-2 were co-expressed (lane 8). This result indicates that the slower migrating band is FLAG–RepoMan conjugated to CFP–SUMO-2, although it is unclear why SUMOylated FLAG–RepoMan migrates much more slowly than expected. Thus, more studies are required to confirm this notion.

Next, the preference of SUMO isoforms for RepoMan modification was examined using HeLa cells expressing CFP, or CFP-tagged SUMO-1, SUMO-2, or SUMO-3 (Fig. 1C). Anti-RepoMan antibody detected a single slower migrating band with an apparent molecular mass of 150 kDa in lysate from HeLa cells expressing CFP-SUMO-2 in addition to the 112 kDa endogenous RepoMan without SUMOylation. Note that a faint signal with similar migration was also detected in lysate containing CFP–SUMO-1 or CFP–SUMO-3. This result provides evidence that endogenous RepoMan can be modified by exogenous SUMOs, especially by SUMO-2 *in vivo.* To address whether RepoMan is SUMOylated *in vivo* and, if so, which isoform is conjugated, untransfected HeLa cell lysates were subjected to immunoprecipitation with an anti-RepoMan antibody followed by western blot analysis (Fig. 1D). Anti-SUMO-2/3 antibody detected a single band with an apparent molecular mass of 125 kDa (lane 9), suggestive of endogenous RepoMan modified by a single molecule of SUMO, whereas we did not detect any bands specifically recognized by anti-SUMO-1 antibody (lane 6). The results provide strong evidence that RepoMan is modified by SUMO-2/3 *in vivo*. Finally, SUMO acceptor sites in the RepoMan polypeptide were then assessed with the GPS-SUMO search tool (http://sumosp.biocuckoo.org/), which predicted two consensus amino acid sequences (residues K627 and K762) in the human RepoMan that had a high probability of SUMOylation. Comparison of amino acid sequences among RepoMan polypeptides of human, mouse, rat, cow, and dog revealed that the candidate IKCE sequence located at amino acid residues 761–764 is highly conserved (Table S1). To test whether K762 acts as a SUMO acceptor, a RepoMan mutant (K762R), with the lysine residue at 762 replaced with arginine, was used for *in vivo* SUMOylation assays (Fig. 1E). When FLAG–RepoMan(WT) was co-expressed with CFP–SUMO-2, a slower migrating band of 250 kDa was detected with the anti-FLAG antibody in addition to the 125 kDa band (lane 3). In contrast, the 250 kDa band was absent in the lysate from cells expressing FLAG–RepoMan(K762R). This result indicates that RepoMan is conjugated by SUMO-2 at the lysine residue at position 762.

### Colocalization and interaction of RepoMan and lamin A on telophase chromosomes

**Fig. 2.**
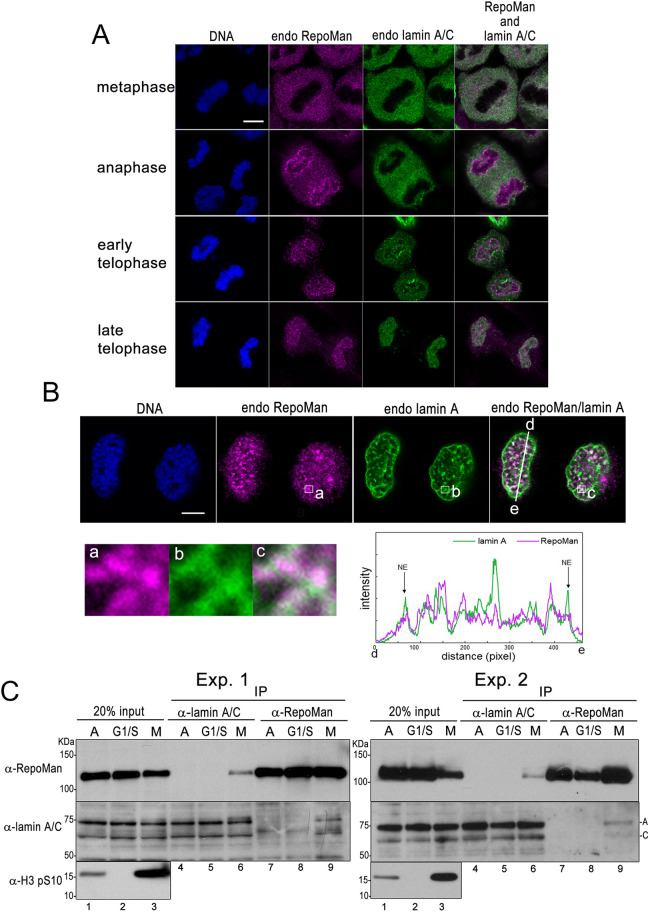
**Colocalizaion and interaction between lamin A and RepoMan during mitosis.** (A) HeLa cells were fixed with 4% formaldehyde in PBS, permeabilized with 0.3% Triton X-100 in PBS and immunofluorescently stained with anti-RepoMan (magenta) and anti-lamin A/C (green) antibodies. DNA was stained with Hoechst 33258 dye (blue). The confocal images depict cells at each stage of mitosis. Scale bar: 5 µm. The images are representative cells examined in two independent experiments (*n*>5 for each mitosis stage). (B) HeLa cells were incubated for 20 h in medium containing 1.5 mM hydroxyurea. At 8 h after release, cells were arrested at the prometaphase by adding monastrol to the medium at the finial concentration of 150 nM for 6 h. Cells were collected by a shaking off method, suspended in 0.5 ml of the medium at 37°C for a further 90 min, and immobilized on a glass slide using cytospinning. After fixation with 4% formaldehyde, telophase cells were immunostained with anti-RepoMan and anti-lamin A/C antibodies. DNA was also stained with Hoechst 33258 dye (blue). The images are representative cells examined in three independent experiments (*n*=6). The colocalizations of two proteins are represented by merged images of green (lamin A) and magenta (RepoMan) immunofluorescence signals. Enlarged images within the white squares (a, b, c) are shown in the lower left panel. The white line (d–e) in the merge image corresponds to the line profile plot shown in the lower right panel. Arrows (NE) indicate the position corresponding to the newly formed nuclear envelope. Scale bar: 5 µm. (C) HeLa cells were arrested at G1/S boundary or M phase by incubating cells in medium containing 1.5 mM hydroxyurea or 1 µg/ml nocodazole for 16 h. The cell lysates were subjected to immunoprecipitation (IP) using an anti-lamin A/C or anti-RepoMan antibody, then proteins were separated on a 5–20% gradient gel by SDS-PAGE and subjected to western blot analysis using anti-lamin A/C and anti-RepoMan antibodies. Synchronization at mitosis with nocodazole treatment was monitored by western blot analysis using antibody specific for anti-mitotic phosphorylation of histone H3 (H3pS10). A, asynchronous; G1/S, hydroxyurea-treated cells; M, nocodazole-treated cells. Blots shown are representative of triplicate experiments.

We next examined the dynamics and colocalization of RepoMan and lamin A during mitosis by immunofluorescence staining. HeLa cells in asynchronous culture were fixed and stained with anti-RepoMan and anti-lamin A/C antibodies (Fig. 2A). Consistent with results in a prior report (Qian et al., 2013), RepoMan was localized in the cytoplasm during metaphase and gathered on the chromosomes at anaphase. Lamin A was similarly distributed in the cytoplasm during metaphase and anaphase, and detected on chromosomes at early telophase. At late telophase, RepoMan and lamin A were apparently colocalized on telophase chromosomes. To further confirm the colocalization, telophase cells spread on a coverslip using the cytospin method were subjected to immunostaining (Fig. 2B). Cells were judged to be at late telophase by assessing chromosome morphology and the existence of prenucleolar bodies according to a previous report (Moriuchi et al., 2016). Confocal microscopy images and plots of fluorescence intensities along the line profile revealed that signals of lamin A and RepoMan considerably overlapped inside of the nuclei in late telophase cells. Notably, colocalization was not observed at the region corresponding to reassembled nuclear lamina, suggesting that lamin A interacts with RepoMan gathering on telophase chromosomes prior to reassembly of nuclear lamina. In order to examine whether RepoMan and lamin A specifically interact during mitosis, co-immunoprecipitation experiments using extracts from asynchronous cultured, G1-S arrested and mitotic arrested HeLa cells were performed. As shown in Fig. 2C, anti-lamin A/C and anti-RepoMan antibodies co-immunoprecipitated considerable amounts of RepoMan (lane 6) and lamin A/C (lane 9) from M phase extract, respectively. In contrast, co-immunoprecipitation of those proteins was scarcely detected in asynchronous and G1/S phase samples. Biochemical data together with immunofluorescence analysis indicate that RepoMan and lamin A specifically associate during mitosis.

### Depletion of RepoMan, a regulatory subunit of protein phosphatase 1γ, delays lamin A reassembly and dephosphorylation of lamin A/C during telophase

**Fig. 3.**
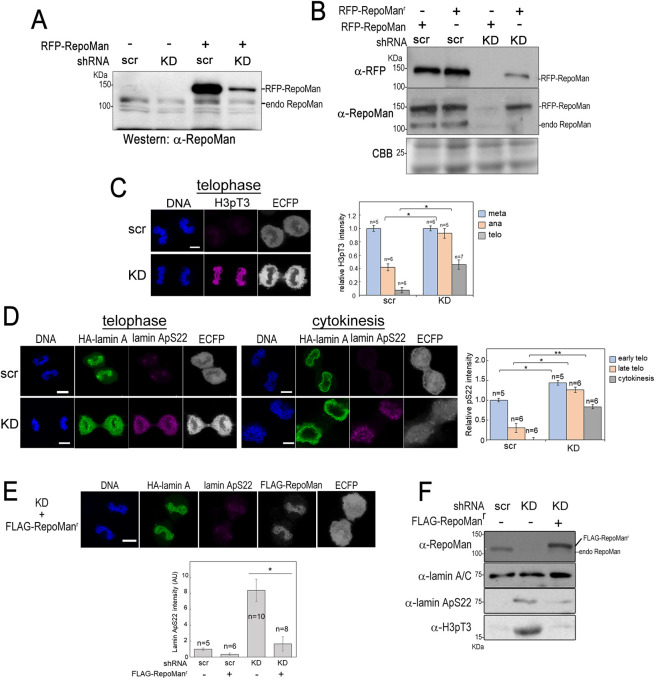
**Knockdown of RepoMan disturbs lamin A reassembly and delays dephosphorylation of lamin A/C.** (A) HeLa cells were transfected with plasmid expressing RFP–RepoMan and plasmid encoding ECFP and scramble (scr) shRNA or shRNA against RepoMan (KD) mRNA. Cells at 24 h after transfection were subjected to western blotting analysis using anti-RepoMan antibody. The transfection efficiency was ∼70% based on numbers of ECFP-positive cells. (B) HeLa cells were transfected with plasmid expressing WT RFP–RepoMan or shRNA-resistant RFP–RepoMan (RFP–RepoMan^r^) and plasmid encoding ECFP and scramble (scr) shRNA or shRNA against RepoMan (KD) mRNA. Cells at 24 h after transfection were subjected to western blotting analysis using anti-RFP antibody. (C) HeLa cells were transfected with shRNA plasmid (scr or KD), then synchronized by thymidine-hydroxyurea-nocodazole block. At 90 min after the release, cells were immunofluorescently stained using anti-H3 pT3 antibody (magenta). The images are representative late telophase cells examined in two independent experiments (scr, *n*=5; KD, *n*=6). Scale bar: 5 µm. The fluorescence signals of H3 pT3 in each ECFP-positive cell at metaphase, anaphase and telophase were quantified and average intensities of signals relative to that of metaphase cells were summarized in the right panel. Data are shown as means±s.e.m. *P<0.05 (Student's *t*-test). (D) HeLa cells were transfected with shRNA plasmid (scr or KD), HA–lamin A plasmid and ECFP plasmid, then synchronized by thymidine-hydroxyurea-nocodazole block. At 90 min after the release, cells were immunofluorescently stained using anti-HA (green) and anti-lamin ApS22 (magenta) antibodies. The images are representative telophase cells (scr, *n*=5; KD, *n*=6) and cells during cytokinesis (scr, *n*=6; KD, *n*=6) examined in two independent experiments. Scale bars: 5 µm. The fluorescence signals of lamin ApS22 in each ECFP-positive cell at metaphase, anaphase and telophase were quantified and expressed relative to the signals of metaphase cells expressing scr shRNA. Data are shown as means±s.e.m. *P<0.05; **P<0.01 (Student's *t*-test). (E) HeLa cells were co-transfected with KD shRNA plasmid, HA–lamin A plasmid and plasmid carrying shRNA-resistant cDNA for FLAG–RepoMan. Cells were synchronized by thymidine-hydroxyurea-nocodazole block. At 90 min after the release, cells were immunofluorescently stained using anti-HA (green), anti-lamin A pS22 (magenta) and anti-FLAG (white) antibodies. The ECFP signal, indicating cells with shRNA plasmid, is also shown. DNA was stained with Hoechst 33258 (blue). Representative images of cell are shown in two independent experiments (*n*=6). Scale bar: 5 µm. The fluorescence signals of lamin A pS22 in each ECFP-positive cell at late telophase were quantified and expressed relative to those of cells transfected with scr shRNA plasmid. Data are shown as means±s.e.m. *P<0.05 (Student's *t*-test). (F) HeLa cells were transfected with scr or KD shRNA plasmid with or without plasmid carrying shRNA-resistant cDNA for FLAG-RepoMan. Cells at 24 h after transfection were subjected to western blotting analysis using anti-RepoMan, anti-lamin A/C, anti-lamin ApS22 and anti-H3 pT3 antibodies. Blots shown are representative of triplicate experiments.

To determine whether depletion of RepoMan affects the dephosphorylation of the mitosis-specific lamin A phosphorylation, we investigated the effect of RepoMan knockdown on lamin A localization on the telophase chromosomes and on dephosphorylation of mitotic-specific lamin A phosphorylation at S22 (lamin A pS22) (Haas and Jost, 1993; Heald and McKeon, 1990). To confirm RNAi efficacy, shRNA against endogenous RepoMan mRNA (KD) or a control shRNA (scramble; scr) and ECFP with or without RepoMan fused with RFP (RFP–RepoMan) were simultaneously expressed in 293FT cells, and signals of endogenous RepoMan and RFP–RepoMan were detected by western blotting analysis using anti-RepoMan antibody. The transfection efficiency was ∼70% based on numbers of ECFP-positive cells. As shown in Fig. 3A, expression of KD shRNA reduced expression levels of both endogenous RepoMan and RFP–RepoMan proteins by more than 60%. In contrast, shRNA-resistant RFP–RepoMan (RFP–RepoMan^r^) was evidently expressed in the cells with KD shRNA (Fig. 3B). Considering the transfection efficiency, we concluded that shRNA markedly reduced RepoMan protein by on-target recognition of RepoMan mRNA. Next, we evaluated functional knockdown of RepoMan protein by detecting signal of phosphorylated Thr3 of histone H3 (H3 pT3), a known substrate of RepoMan–PP1γ (Qian et al., 2011; Vagnarelli et al., 2011) (Fig. 3C). The H3 pT3 signals were reduced from metaphase to telophase by 93% in control, whereas 45% of signals remained in telophase cells expressing RepoMan shRNA. This result indicates that KD shRNA results in significant loss of RepoMan function in mitotic cells.

Next, HeLa cells were simultaneously transfected with HA–lamin A and shRNA plasmids, then synchronized at early M phase via a thymidine-hydroxyurea-nocodazole block. Cells at 90 min after the release from nocodazole were subjected to immunofluorescence staining with anti-HA and anti-lamin A/C pS22 antibodies (Fig. 3D). Normal accumulation and dephosphorylation of HA–lamin A on telophase chromosome were clearly observed in cells expressing scr shRNA. Meanwhile, RepoMan depletion delayed or inhibited HA–lamin A accumulation on telophase chromosomes and the subsequent lamina formation. Quantification of immunofluorescence showed that only 41% of the lamin A pS22 was dephosphorylated from metaphase to cytokinesis in RepoMan-depleted cells. It should be noted that signals of another mitotic phosphorylation of lamin A/C, at Ser392 (laminA/C p392), were similarly retained in RepoMan-depleted cells during late telophase and after cell division (Fig. S1). The abnormalities caused by RepoMan depletion mutant are quite similar to those observed in the lamin A SIM3, as described previously (Moriuchi et al., 2016). Considering that SIM3 mutation delayed the dephosphorylation of mitosis-specific phosphorylated lamin A, we predicted that RepoMan depletion caused the delay of lamin A dephosphorylation.

To confirm whether the observed phenotype was specifically induced by depletion of RepoMan protein, we next performed rescue experiments using a plasmid expressing RNAi-resistant RepoMan. The defects of lamin A assembly to the telophase chromosome and lamin A pS22 dephosphorylation, along with the abnormal nuclear lamina morphology caused by RepoMan knockdown were rescued by the expression of RepoMan^r^ expression (Fig. 3E,F). These results strongly suggest that the RepoMan is involved in the proper dephosphorylation of lamin A pS22 during telophase and nuclear lamina formation.

### SUMO acceptor K762 of RepoMan is required for dephosphorylation of lamin A and nuclear lamina formation

**Fig. 4.**
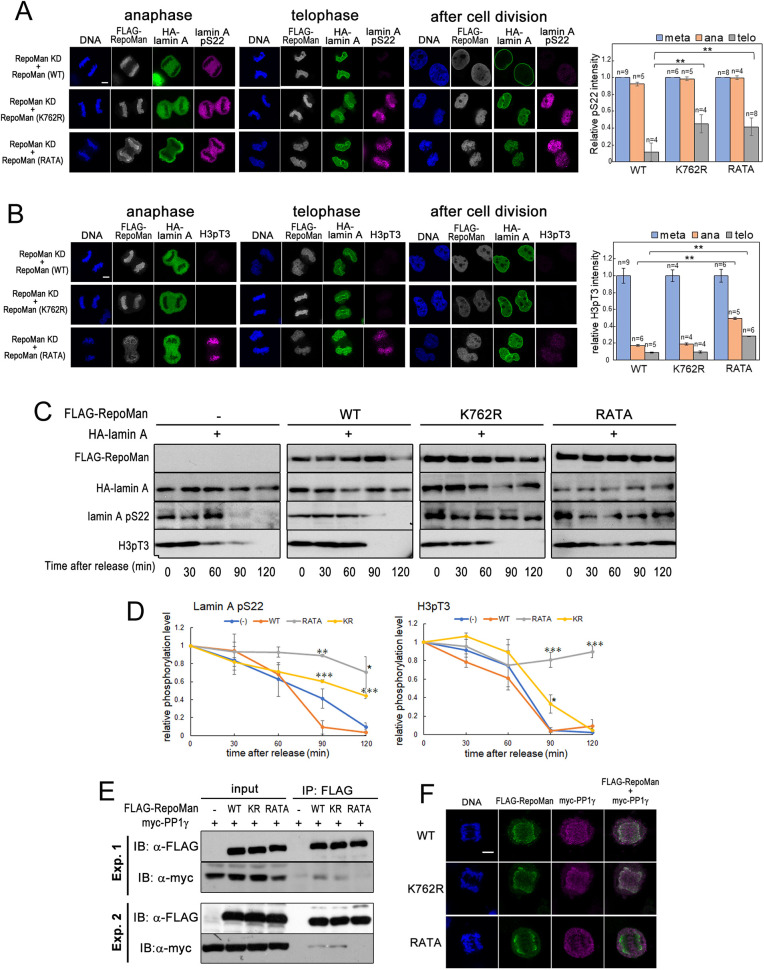
**The SUMOylated RepoMan–PP1 holoenzyme is likely responsible for dephosphorylation of lamin A.** HeLa cells were transfected with shRNA plasmid for RepoMan knockdown (KD), FLAG–RepoMan plasmid encoding shRNA-resistant RepoMan mRNA (WT, K762R mutant or RATA mutant) and HA–lamin A plasmid as indicated and synchronized by the hydroxyurea-nocodazole method. (A,B) Cells were fixed with 4% formaldehyde in PBS at 60, 75 and 90 min after release from the nocodazole blockage and immunostained with anti-FLAG (white), anti-HA (green), anti-lamin ApS22 (magenta) antibodies (A), or immunostained with anti-FLAG (white), anti-HA (green), and anti-H3 pT3 (magenta) antibodies (B). DNA was stained with the Hoechst 33258 (blue). Representative images of cells (of more than four cells for each mitosis stage) are shown from two independent experiments. Scale bars: 5 µm. The fluorescence intensities of lamin A pS22 or H3 pT3 signals of each cell at metaphase (meta), anaphase (ana) and telophase (telo) were quantified and expressed relative to those of metaphase cells. Data are shown as means±s.e.m. **P<0.01 (Student's *t*-test). (C) HeLa cells transiently expressing FLAG–RepoMan (WT, K762R mutant or RATA mutant) and HA–lamin A were collected at the indicated time point after release from the hydroxyurea-nocodazole blockage and directly lysed in Laemmli's sample buffer. The samples were then subjected to SDS-PAGE and proteins were detected by western blot analysis using anti-FLAG, anti-HA, anti-lamin ApS22 and anti-H3 pT3 antibodies. (D) Quantification of lamin ApS22 and H3 pT3 signals after the release from the nocodazole blockage. Data are mean±s.e.m. and represent three independent experiments. *P<0.05; **P<0.01; ***P<0.001 versus WT RepoMan transfection (Student's *t*-test). (E) 293FT cells were simultaneously transfected with plasmids expressing FLAG–RepoMan (WT, K762R, or RATA) and Myc–PP1γ as indicated. The cell lysates were subjected to immunoprecipitation (IP) using an anti-FLAG antibody, then proteins were separated by SDS-PAGE and detected by western blotting using anti-FLAG and anti-Myc antibodies. Input represents 25% of lysate. Blot shown is representative of three experiments. (F) HeLa cells were simultaneously transfected with plasmids expressing FLAG–RepoMan (WT, K762R or RATA) and Myc–PP1γ. At 24 h after transfection, cells were fixed and stained using anti-FLAG (green) and anti-Myc (magenta) antibodies. DNA was stained with Hoechst 33258 (blue). Scale bar: 5 µm. Representative images of anaphase cells are shown from two independent experiments (WT, *n*=4; K762R, *n*=3; RATA, *n*=3).

To elucidate whether SUMOylation of RepoMan and RepoMan-associated PP1γ are required for dephosphorylation of lamin A pS22 and nuclear lamina formation, the FLAG–RepoMan K762R mutant or FLAG–RepoMan RATA mutant, which has amino acid substitutions in its PP1-binding RVxF motif (Trinkle-Mulcahy et al., 2006), was co-expressed with HA–lamin A in HeLa cells in which the expression of endogenous RepoMan was transiently knocked down. As shown in Fig. 4A,B, the effects of mutant RepoMan expression on lamin A localization and dephosphorylation of lamin A pS22 and H3 pT3 during mitosis were examined by immunofluorescence staining. Localization patterns of K762R and RATA mutants were almost similar to that of WT RepoMan through anaphase, telophase and cytokinesis. During anaphase, no difference was observed in HA–lamin A localization among cells expressing WT RepoMan, the RATA mutant or the K762R mutant. In telophase, HA–lamin A in cells expressing WT RepoMan localized normally to the region surrounding the telophase chromosomes, and was subsequently dephosphorylated and reassembled into nuclear lamina. In contrast, K762R mutant expression resulted in an incomplete accumulation of HA–lamin A around the telophase chromosome, and quantification of the fluorescence signals showed marked inhibition of lamin A pS22 dephosphorylation in telophase cells. Expression of the RATA mutant similarly delayed dephosphorylation of lamin A pS22, although HA–lamin A accumulation on telophase chromosomes occurred normally. Notably, strong H3 pT3 signals were detected in telophase cell expressing the RATA mutant, whereas such signals were only detected at very low level in cells expressing WT or K762R RepoMan, indicating that K762R RepoMan does not delay the dephosphorylation of H3 pT3 (Fig. 4B). These results indicate that the association of RepoMan with PP1γ is required for the dephosphorylation of both lamin A pS22 and H3 pT3. Moreover, it should be emphasized that the SUMO acceptor lysine at 762 is required for the dephosphorylation of lamin A pS22 but not that of H3 pT3.

Next, we quantitatively evaluated the effect of RepoMan mutant expression on the dephosphorylation of lamin ApS22 and H3 pT3 during mitosis by western blot analysis using lysates from cells released from hydroxyurea-nocodazole synchronized cells (Fig. 4C,D). However, we observed that simultaneous expression of K762R or RATA mutant in RepoMan shRNA-expressing cells severely impaired normal progression of mitosis after release from nocodazole block. Therefore, we used HeLa cells expressing FLAG–RepoMan (WT, K762R or RATA) and HA–lamin A without RepoMan knockdown in the analysis. In control cells or HeLa cells with WT RepoMan expression, lamin ApS22 and H3 pT3 were similarly dephosphorylated at ∼90 min after the release from nocodazole block. In contrast, expression of either the K762R or RATA mutant significantly inhibited lamin A pS22 dephosphorylation. In comparison, the K762R mutant did not inhibit dephosphorylation of H3 pT3 whereas RATA mutant completely inhibited it. These results further support our hypothesis that SUMOylation at K762 of RepoMan is required for the dephosphorylation of lamin ApS22.

RepoMan has been identified as a specific regulatory subunit for PP1γ and plays a role of targeting PP1γ to the chromosome in anaphase. Hence, the possibility exists that the defect in lamin A dephosphorylation seen upon K762R RepoMan expression may be caused by the loss of RepoMan interaction with PP1γ and recruitment of PP1γ to anaphase chromosomes. To address this question, we performed immunoprecipitation experiments and immunostaining using cells expressing FLAG–RepoMan and Myc–PP1γ (tagging of the catalytic subunit). The results showed that the K762R mutant was bound to PP1γ (Fig. 4E) and could recruit PP1γ to chromosomes after anaphase onset in a similar manner to the WT RepoMan (Fig. 4F). Therefore, we conclude that RepoMan SUMOylation at K762 is responsible for the accumulation on the telophase chromosome, timely dephosphorylation and nuclear lamina formation of lamin A.

### SUMOylation of RepoMan enhances interaction with lamin A

**Fig. 5.**
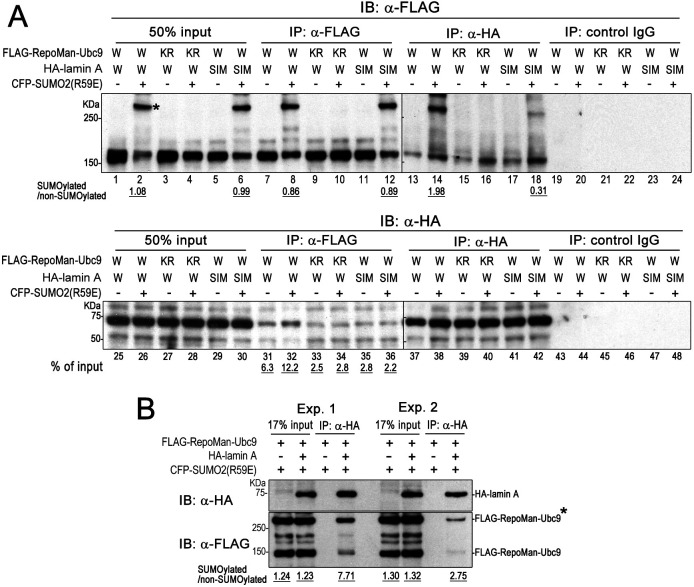
**SUMOylation of RepoMan enhances the interaction between RepoMan and lamin A.** (A) 293FT cells were transfected with plasmids expressing HA–lamin A(WT) (W) or HA–lamin A[SIM3(AA)] (SIM), FLAG–RepoMan(WT)–Ubc9 (W) or FLAG-RepoMan(K762R)-Ubc9 (KR), and CFP–SUMO-2(R59E) as indicated and subjected to immunoprecipitation (IP) using an anti-FLAG antibody, anti-HA antibody or normal mouse IgG. FLAG–RepoMan with or without SUMOylation and HA–lamin A in input and immunoprecipitated samples were detected using anti-FLAG and anti-HA antibodies, respectively. The asterisk indicates the CFP–SUMO-2 modified FLAG–RepoMan–Ubc9. Relative amounts of SUMOylated RepoMan and non-SUMOylated RepoMan in each sample were determined by quantifying the signals of immunoblots (IB) using ImageJ. The amounts of HA–lamin A precipitated using anti-FLAG antibody were quantified from signals of triplicate experiments. (B) Quantification of RepoMan with or without SUMOylation in immunoprecipitation samples. 293FT cells were transfected with the indicated combinations of expression plasmids and subjected to immunoprecipitation using anti-HA antibody. HA–lamin A and FLAG–RepoMan in input and immunoprecipitated samples were detected using anti-HA and anti-FLAG antibodies, respectively. The asterisk indicates the CFP–SUMO-2 modified FLAG–RepoMan–Ubc9. Relative amounts of SUMOylated RepoMan and non-SUMOylated RepoMan in each sample were determined by quantifying the signals of immunoblots using ImageJ. Blots shown are representative of triplicate experiments.

To address whether SUMOylation of RepoMan affects the specificity or affinity to lamin A and whether the SIM3 domain of lamin A at amino acid residues 494–497 (VVTI) is required for the interaction with RepoMan, an immunoprecipitation experiment was performed using a RepoMan mutant (K762R) which is defective for SUMO conjugation and a lamin A mutant [SIM3(EE)] in which two hydrophobic amino acids (V494, V495) were replaced by glutamic acid (Moriuchi et al., 2016). 293FT cells were transfected with HA–lamin A, CFP–SUMO-2 and FLAG–RepoMan plasmids in various combinations and subjected to immunoprecipitation using anti-HA and anti-FLAG antibodies. However, we noticed that SUMOylated species of RepoMan were markedly diminished during the immunoprecipitation procedure, which may be attributed to the presence of SUMO isopeptidases in cell lysates. Consequently, we used a SUMO-2 mutant (R59E) that can be conjugated to substrate proteins but is resistant to SUMO isopeptidase activity as it lacks an amino acid residue (R59) required for SUMO isopeptidase recognition (Ihara et al., 2007). In addition, we sought to use the Ubc9 fusion-directed SUMOylation system, which is widely applied to mediate conjugation of SUMO to substrate proteins. Lysates of 293FT cells expressing HA–lamin A [WT or SIM3(EE)], CFP or CFP–SUMO-2(R59E), and FLAG–RepoMan(WT)–Ubc9 or FLAG–RepoMan(K762R)–Ubc9 were subjected to immunoprecipitation (Fig. 5A). As evident in the input sample, co-expression of CFP–SUMO-2(R59E) and FLAG–RepoMan(WT)-Ubc9 resulted in marked enhancement of RepoMan SUMOylation (lane 2). Immunoprecipitation using anti-FLAG antibody revealed that WT RepoMan with CFP–SUMO-2 expression precipitated WT HA–lamin A most effectively (lane 32). As expected, KR mutation of RepoMan (lanes 33 and 34) and SIM mutation in lamin A (lanes 35 and 36) significantly weakened binding between lamin A and RepoMan. Reciprocal immunoprecipitation using anti-HA antibody also showed association of HA–lamin A with WT RepoMan. Notably, quantification of the relative binding to SUMOylated and non-SUMOylated RepoMan revealed that WT lamin A binds preferentially to SUMOylated RepoMan (lane 14). However, the SIM3(EE) mutant did not exhibit such a preference for association with SUMOylated RepoMan (lane 18). This preference was confirmed by two independent immunoprecipitation experiments (Fig. 5B). These results indicate that SUMOylation of RepoMan evidently enhances its binding affinity with lamin A and that SIM3 of lamin A is critical for facilitating specific interaction between the two molecules.

### Lamin A and RepoMan associate via SUMO–SIM interaction

**Fig. 6.**
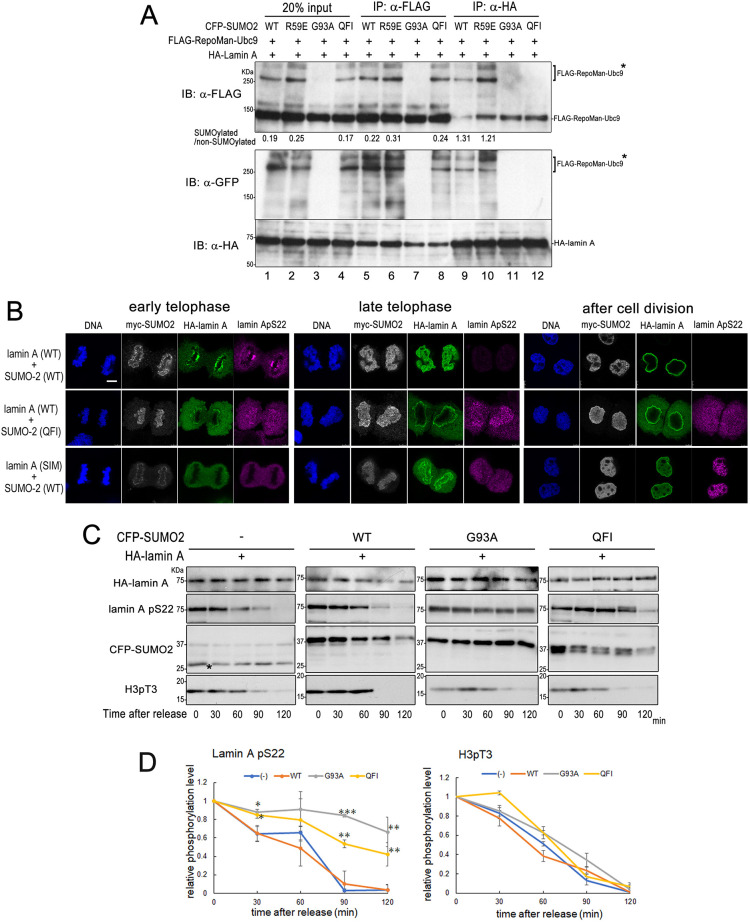
**SUMO–SIM interaction is required for interaction between lamin A and RepoMan and also dephosphorylation of lamin A.** (A) 293FT cells were transfected with expression plasmids for CFP–SUMO-2 (WT, R59E, G93A or QFI), FLAG–RepoMan–Ubc9 and HA–lamin A as indicated. At 24 h after DNA transfection, cells were lysed and subjected to immunoprecipitation using an anti-FLAG or anti-HA antibody. Proteins were detected by western blot analysis using anti-FLAG, anti-HA and anti-GFP antibodies. The asterisks indicate the CFP–SUMO-2-modified FLAG–RepoMan–Ubc9. The values represent relative amount of SUMOylated and non-SUMOylated FLAG–RepoMan–Ubc9, which were derived from signals of triplicate immuoblots. (B) HeLa cells were simultaneously transfected with Myc–SUMO-2 (WT or QFI) and HA–lamin A [WT or SIM3(EE)] expression plasmids. At 36 h after DNA transfection, cells were fixed with 4% formaldehyde in PBS and immunofluorescently stained with anti-myc (white), anti-HA (green), and anti-lamin Ap22 (magenta) antibodies. DNA was stained with Hoechst 33258 dye (blue). The confocal images depict cells at each stage of mitosis. Representative images of cells (more than four cells for each mitosis stage) are shown from two independent experiments. Scale bar: 5 µm. (C) HeLa cells transiently expressing HA–lamin A and CFP–SUMO-2 (WT, G93A or QFI) were synchronized by the hydroxyurea-nocodazole method. Cells were collected at the indicated time point after release from the nocodazole blockage and directly lysed in Laemmli's sample buffer. The samples were then subjected to SDS-PAGE and proteins were detected by western blot analysis using anti-HA, anti-lamin A pS22, anti-H3 pT3, and anti-GFP antibodies. (D) Quantification of lamin ApS22 and H3 pT3 signals after the release from the nocodazole blockage. Data are mean±s.e.m.; *n*=3 independent experiments. *P<0.05; **P<0.01; ***P<0.001 versus WT SUMO-2 expression (Student's *t*-test).

We next sought to directly test the requirement for the SUMO–SIM interaction between RepoMan and lamin A for the dephosphorylation of lamin A. For this purpose, we used a SUMO-2 QFI mutant in which hydrophobic amino acids (Q35, F36, and I38) within the second β-strand of SUMO-2, known to be responsible for SIM interaction were replaced by alanine. Overexpression of QFI mutant has been shown to impair SUMO–SIM interaction in a dominant-negative manner (Hecker et al., 2006; Merrill et al., 2010). 293FT cells were transfected with FLAG–RepoMan–Ubc9, HA–lamin A and WT or any single SUMO-2 mutant (R59E for extensive SUMOylation, G93A for inhibition of SUMOylation, or QFI for inhibition of SUMO–SIM interaction) and subjected to immunoprecipitation using anti-FLAG and anti-HA antibodies (Fig. 6A). Western blot analysis of input samples with an anti-FLAG and anti-GFP antibodies revealed that RepoMan is obviously SUMOylated with WT (lane 1), R59E (lane 2) and QFI SUMO-2 (lane 4), although there is no conjugation with G93A SUMO-2 (lane 3). Consistent with results shown in Fig. 5, HA–lamin A preferentially precipitated SUMOylated compared to non-SUMOylated RepoMan when using cell lysate containing the WT or R59E SUMO-2 (lanes 9 and 10). Using cell lysate with QFI SUMO-2, HA–lamin A precipitated only RepoMan species without SUMOylation (lane 12), indicating the possibility that RepoMan conjugated with QFI SUMO-2 impairs the interaction between RepoMan and lamin A. Taken together, we conclude that lamin A specifically binds to RepoMan via a SUMO–SIM interaction.

We further examined the effect of the QFI mutant on the localization and dephosphorylation process of lamin A during mitosis. HeLa cells were transfected with Myc–SUMO-2 (WT) or Myc–SUMO-2(QFI) plasmid and HA–lamin A [WT or SIM3(EE)] plasmid. At 36 h after DNA transfection, cells were immunofluorescently stained using anti-myc, anti-HA, and anti-lamin A/C pS22 antibodies. As shown in Fig. 6B, expression of Myc–SUMO-2(QFI) abrogated lamin A reassembly at late telophase and caused a disordered nuclear lamina. Furthermore, signals of lamin A pS22 were observed on the late telophase chromosomes in cells with SUMO-2(QFI) whereas these had almost disappeared in the late telophase cells transfected with SUMO-2(WT). Moreover, signals of lamin A pS22 remained in all populations of SUMO-2(QFI)-expressing cells after cell division. Similarly, lamin A pS22 signals were observed in cells expressing HA–lamin A SIM3(EE) at late telophase and after cell division, suggesting again that the SIM3 of lamin A is required for normal dephosphorylation of lamin A pS22.

To further confirm the immunostaining data, we next quantified changes in the levels of lamin A pS22 during mitosis by immunoblot analysis (Fig. 6C,D). For these analyses, HeLa cells were transfected with plasmids expressing WT HA–lamin A and the WT CFP–SUMO-2 or its mutant forms (G93A or QFI), then synchronized at the beginning of mitosis by thymidine-hydroxyurea-nocodazole arrest. Cells were collected by shaking, transferred to fresh medium, harvested and lysed at various time points. We also examined dephosphorylation of H3 pT3, a reported target of RepoMan–PP1γ. Expression of SUMO-2(QFI) as well as SUMO-2(G93A) delayed the dephosphorylation of lamin A pS22. Conversely, both SUMO-2 mutants did not delay the dephosphorylation of H3 pT3, suggesting that the SUMO–SIM interaction is needed for the dephosphorylation of laminA pS22 but not that of H3 pT3.

### RepoMan is transiently SUMOylated at telophase

**Fig. 7.**
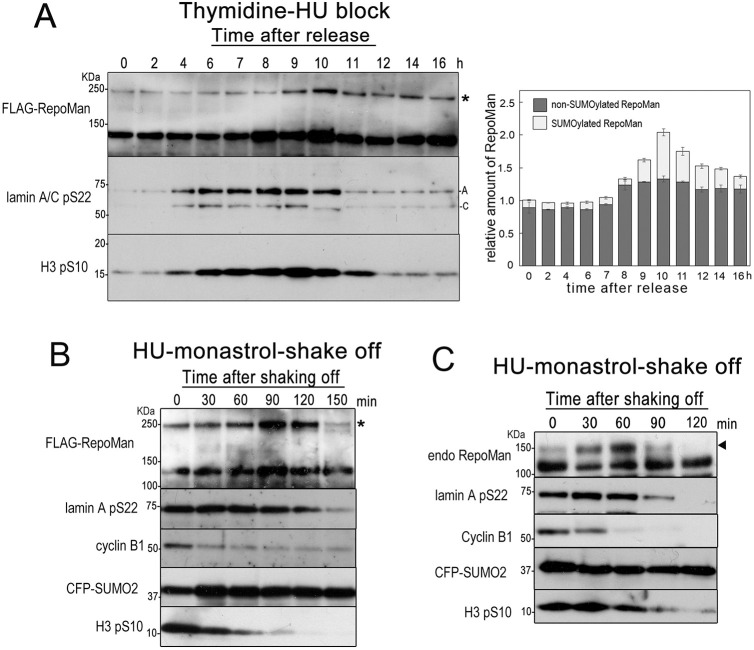
**RepoMan SUMOylation peaks during telophase.** (A) HeLa cells transiently expressing FLAG–RepoMan and CFP–SUMO-2(WT) were synchronized by the thymidine-hydroxyurea method. Cells were collected at the indicated time point after release from the thymidine blockage and directly lysed in Laemmli's sample buffer. The samples were then subjected to SDS-PAGE and proteins were detected by western blot analysis using anti-FLAG, anti-lamin A/C pS22, and anti-H3 pS10 antibodies. The asterisk, A and C indicate the CFP–SUMO-2-modified FLAG–RepoMan, lamin A and lamin C, respectively. The data is representative of three independent experiments with similar results. Amounts of FLAG-RepoMan with or without SUMOylation were determined by quantifying the signals of immunoblots using ImageJ and summarized in the graph. Data are mean±s.e.m. and represent three independent experiments. (B) HeLa cells transiently expressing CFP–SUMO-2(WT) were synchronized by the hydroxyurea-monastrol method. Prometaphase cells were collected by shake-off, seeded and cultured in 12-well plates for the indicated time. Cells were then lysed in Laemmli's sample buffer. The samples were then subjected to SDS-PAGE and proteins were detected by western blot analysis using anti-FLAG, anti-lamin A/C pS22, anti-H3 pS10, anti-cyclin B and anti-GFP antibodies. The asterisk indicates the CFP–SUMO-2-modified FLAG–RepoMan. The data is representative of two independent experiments with similar results. (C) HeLa cells transiently expressing CFP–SUMO-2(WT) were synchronized and processed as in B. The arrowhead indicates endogenous RepoMan modified with CFP–SUMO-2. The data is representative of two independent experiments with similar results.

Shimmel et al. have performed proteomics analysis of SUMOylated proteins purified from G2/M phase cells arrested by RO-3306, an inhibitor of Cdk1 (Schimmel et al., 2014). Shou et al. also have shown the results of proteomics of proteins conjugated with SUMO-2/3 using M phase cells obtained by thymidine-taxol double block (Schou et al., 2014). From their studies, RepoMan was found to be one of the proteins conjugated by SUMO-2/3 in an M-phase specific manner. However, the detail of fluctuation of RepoMan SUMOylation during cell cycle progression was not clear. Therefore, HeLa cells were synchronized and the SUMOylation status of RepoMan was investigated. Cells expressing FLAG–RepoMan and CFP–SUMO-2(WT) were arrested at the G1/S boundary by thymidine-hydroxyurea double block and cells at different time points (0–16 h) after the release from hydroxyurea were used for western blot analysis (Fig. 7A). Entrance and exit of mitosis were monitored by western blot analysis using anti-histone H3 phosphorylated at Ser10 (H3 pS10) antibody (Hendzel et al., 1997). Signals of H3 pS10 indicated that the major population of cells entered M phase at ∼6–7 h and exited at 11 h after release. The fluctuations of SUMOylated FLAG–RepoMan and lamin A pS22 were probed with anti-FLAG and anti-lamin A/C pS22-specific antibodies using the same blot. Dephosphorylation of lamin A/C pS22 occurred at telophase just after that of H3 pS10, confirming the onset of lamin A dephosphorylation at the later part of telophase. Total amount of FLAG–RepoMan was increased by two-fold during the period coincident with mitosis. Notably, a significant elevation of RepoMan SUMOylation occurred during short period of mitosis. The relative amount of SUMOylated FLAG–RepoMan was constant by 8 h after release (∼10% of total FLAG–RepoMan), rapidly increased from 8 h after release and peaked at 10 h after release (38% of total FLAG–RepoMan), although lamin A/C pS22 signals remained. To more precisely clarify the timing of RepoMan SUMOylation during mitosis, we synchronized HeLa cells transfected with the CFP–SUMO-2 and FLAG–RepoMan expression plasmids using hydroxyurea-monastrol block. The prometaphase cells collected by shake-off were incubated in fresh medium for the indicated periods and subjected to western blot analysis (Fig. 7B). Fluctuations of cyclin B1, H3 pS10 and lamin A pS22 implied successful cell cycle synchronization and mitosis progression after release. Under these conditions, anti-FLAG antibody detected a transient increase and decrease of SUMOylated FLAG–RepoMan at 90 and 150 min after release from monastrol, respectively. We also examined the timing of endogenous RepoMan SUMOylation using cells expressing CFP–SUMO-2 (Fig. 7C). Similarly, the signal of endogenous SUMOylated RepoMan exhibited a transient increase and decrease, with a peak at 60 min after release. Observation of cell morphology using microscopy revealed that the proportion of telophase cells peaked from 60 to 90 min after release (Fig. S2). These results indicate that RepoMan is transiently SUMOylated at the later part of telophase, prior to the occurrence of lamin A pS22 dephosphorylation.

## DISCUSSION

For human lamin A, phosphorylation at Ser22 and Ser392 by Cdk1–Cyclin B, often referred to as mitosis-specific phosphorylation, triggers disassembly of the nuclear lamina by inhibiting polymerization of the lamin A dimer (Heald and McKeon, 1990; Peter et al., 1990). This mitotic phosphorylation of lamin A is maintained until the beginning of telophase, which might be significant for reorganizing the nuclear envelope and ensuring a proper connection between the nuclear lamina and chromatin at the end of mitosis. However, the factor(s) and regulatory mechanisms controlling the dephosphorylation of lamin A and nuclear lamina reconstitution were not fully understood. Our previous study suggested that unknown SUMOylated factor(s) localized on the telophase chromosome were responsible for chromosomal accumulation and timely dephosphorylation of lamin A (Moriuchi et al., 2016). The present study was thus performed to identify and characterize the putative SUMOylated factor(s) involved in lamin A dephosphorylation.

We have shown that SUMOylation of RepoMan plays a crucial role in the recruitment of phosphorylated lamin A to telophase chromosomes, dephosphorylation of mitosis-specific lamin A phosphorylation and the subsequent nuclear lamina formation. We have also demonstrated that K762 of RepoMan is transiently SUMOylated on the chromosomes during the latter part of telophase. Immunoprecipitation experiments revealed that SUMOylation of RepoMan enhances its binding affinity with lamin A. Expression of the lamin A mutant without a functional SIM, a RepoMan mutant without the SUMO acceptor K762 and a SUMO-2 mutant defective in binding to the SIM all resulted in impairment of the interaction between RepoMan and lamin A, accumulation of lamin A on telophase chromosomes, dephosphorylation of lamin A at the end of mitosis and nuclear lamina formation. Together, these findings indicate that the transient SUMOylation of RepoMan crucially controls the sequential process including the recruitment of lamin A to telophase chromosomes and its dephosphorylation, along with the subsequent nuclear lamina formation.

The PP1γ–RepoMan holoenzyme has already been confirmed as an essential phosphatase for mitotic exit (Qian et al., 2011; Vagnarelli et al., 2011). Recent investigations have demonstrated that the phosphorylation states of specific serine or threonine residues on the RepoMan polypeptide mediated by Cdk1–cyclin B changes the RepoMan-associated phosphatase from PP2A to PP1 (Qian et al., 2015). At the onset of anaphase, degradation of Cdk1–cyclin B results in decreased RepoMan phosphorylation at T232 and S893, thereby enriching the amount of RepoMan–PP1γ holoenzyme that possesses a higher affinity to the chromosome and triggering dephosphorylation of histone H3 at T3, S10 and S28 at the beginning of telophase (Vagnarelli et al., 2011). However, we observed that dephosphorylation of lamin A apparently begins 30 min later than that of histone H3, suggesting that chromosome binding of RepoMan–PP1γ is not sufficient to mediate lamin A dephosphorylation. Accordingly, we suggest that the transient SUMOylation of RepoMan during the latter part of telophase plays an essential role in defining the timing of lamin A dephosphorylation. To confirm this conjecture, it is necessary to clarify how RepoMan is transiently SUMOylated. In general, the balance between SUMO conjugation and SUMO deconjugation enzymes controls the SUMOylation level of substrates. To investigate whether this is the case here, it would be necessary to identify the E3 ligases and SUMO isopeptidase that specifically modulate the SUMOylation level of RepoMan. In future studies, we are planning to test several candidates, including RanBP2 and PIAS family members, which are known as chromosome-associated E3 ligases, along with several SENPs that have been demonstrated to regulate the deSUMOylation of specific substrates required for mitotic events (Mukhopadhyay and Dasso, 2017). Among these candidates, we expect that RanBP2 is the most probable E3 ligase for RepoMan SUMOylation. RanBP2, which is the nucleoporin that is localized at the cytoplasmic periphery of the nuclear complex, serves an essential function in nucleocytoplasmic transport via interaction with importin β during interphase (Wu et al., 1995). After nuclear envelope breakdown and nuclear pore complex disassembly, RanBP2 is localized to mitotic microtubules and kinetochore after microtubules attachment (Joseph et al., 2002) and SUMOylates proteins important for chromosome segregation during metaphase to anaphase (Dawlaty et al., 2008; Werner et al., 2012). Previous studies have demonstrated that the spatiotemporal pattern of RanBP2 in mitosis depends on interactions with importin β and CRM1 (also known as exportin 1) (Gilistro et al., 2017; Roscioli et al., 2012). Interestingly, Vagnarelli et al. have found that RepoMan interacts directly with importin β and targets a fraction of the importin β content to the chromosome periphery at the end of anaphase (Vagnarelli et al., 2011). Considering the specific association between importin β and RanBP2 during mitosis, it is possible that RanBP2 is recruited to the chromosome periphery later in anaphase through indirect interaction with RepoMan via the intermediation of importin β and transiently SUMOylates RepoMan.

We further hypothesized that the accessibility of E3 SUMO ligase to RepoMan may control the periodical SUMOylation of RepoMan. The RepoMan–PP1γ holoenzyme targeted to chromosomes at anaphase dephosphorylates mitotic phosphorylation at T3, S10 and S28 of histone H3. A recent study has revealed that dephosphorylation of histone H3 at T3 and S10 and dephosphorylation of histone H3 at S28 by RepoMan–PP1γ allow the re-association of HP1 at histone H3 trimethylated at K9 and histone H3 trimethylated at K27, respectively, indicating that dynamic changes in chromatin conformation caused by histone H3 dephosphorylation might lead to sequential events (de Castro et al., 2017). This raises a possibility that dephosphorylation of histone H3 is relevant to the accessibility of an E3 ligase to RepoMan. Although we have no evidence supporting this hypothesis, conformational change of chromatin triggered by histone H3 dephosphorylation might allow RepoMan SUMOylation. To examine this possibility, identification of the E3 ligase along with live imaging of the various players during telophase should be performed.

In conclusion, we have demonstrated that phosphorylated lamin A associates with telophase chromosomes via an interaction between its SIM3 and the SUMOylated RepoMan–PP1γ chromatin-binding holoenzyme, after which lamin A is dephosphorylated. Moreover, this SUMO–SIM interaction facilitates the reassembly of the nuclear lamina around the daughter chromosomes. Thus, our findings provide novel insights into this important process by revealing critical features of the SUMO–SIM interaction that allows temporal and spatial regulation of the ordered events at the end of mitosis.

## MATERIALS AND METHODS

### Plasmids

The plasmids used for mammalian expression of HA-tagged WT and lamin A SIM3 mutants were described previously (Moriuchi et al., 2016). The RepoMan WT plasmid was generated by inserting the respective human RepoMan cDNA molecules into the pCSII-hMTIIA(ΔGRE)-HA-IRES2-Venus or pCSII-hMTIIA(ΔGRE)-FLAG-IRES2-Venus lentivirus vector containing the human metallothionein-IIA promoter, as described previously (Fumoto et al., 2007). In addition, base substitution mutations (K762R and RATA) and an shRNA-resistant mutation (RepoMan^r^) were introduced into the RepoMan cDNA via site-specific mutagenesis using the overlap extension method with the following primers, and confirmed by DNA sequencing: RepoMan (K762R), 5′-AACATAAGGTGTGAAAGAAAGGATGA-3′ and 5′-GTGGTCTAAATTTGTATTCCACACTT-3′; RepoMan (RATA), 5′-AGGAAGAGAGCTACTGCTGGAGAGGACTTAAG-3′ and 5′-CCTTCTCCTTCTCTCGATGACGACCTCTCCTG-3′; RepoMan (K762R), 5′-AACATAAGGTGTGAAAGAAAGGATGA-3′ and 5′-GTGGTCTAAATTTGTATTCCACACTT-3′; and RepoMan^r^, 5′-CCGATCTCACACGCAAGGAAGGTCTCAGCGCT-3′ and 5′-TTGCGTGTGAGATCGGTCATCTCGGACTCTTTT-3′.

The expression plasmid for the RepoMan–Ubc9 fusion protein was created by overlap extension PCR as follows. The cDNAs for RepoMan and Ubc9 were amplified by PCR using each pair of primers; RepoMan (without stop codon), 5′-TTACTCGAGGATGCCAATTCAAAAGACAA-3′ and 5′-ACCTCCGCTCCCGCCCTGCTTTCTTTCTCCATTAT-3′; and *UBC9* (without initiation codon), 5′-GGCGGGAGCGGAGGTTCGGGGATCGCCCTCAGCAG-3′ and 5′-CCTGGATCCTTATGAGGGCGCAAACTTCTTGG-3′ (overlapping sequences that encode the N-terminal amino acids of Ubc9 are underlined). Two PCR products were mixed, and the second PCR was performed with primers that produce a DNA fragment encoding the RepoMan–Ubc9 fusion protein. Finally, the resultant DNA fragment was digested with XhoI (TAKARA Bio Inc, Kyoto, Japan) and BamHI (TAKARA Bio Inc, Kyoto, Japan) and cloned into the compatible sites of the pCSII-hMTIIA(ΔGRE)-FLAG-IRES2-Venus vector.

The plasmid expressing shRNA against endogenous RepoMan mRNA was prepared as follows. A pair of oligonucleotides containing a 19-nucleotide targeting sequence (5′-TGACAGACTTGACCAGAAA-3′) was annealed and cloned into an attL-containing pENTR/U6 plasmid (Thermo Fisher Scientific); the oligonucleotides were as follows: 5′-CACCGTGACAGACTTGACCAGAAACGAATTTCTGGTCAAGTCTGTCA-3′ and 5′-AAAATGACAGACTTGACCAGAAATTCGTTTCTGGTCAAGTCTGTCAC-3′. The cassette containing the U6 promoter and shRNA target sequence was then transferred to a plasmid (pCS-RfA-EC) carrying *ECFP* cDNA under the control of the EF-1α promoter. pCS-RfA-EC plasmid was created by replacing cDNA for GFP on pCS-RfA-EG (RIKEN BRC DNA Bank) with that for ECFP from pECFP-C1 (TAKARA Bio USA, Inc.).

Expression constructs of the WT and G93A SUMO2 with a Myc tag or fused with CFP were generated as described previously (Yamashita et al., 2004). The SUMO2 (R59E and QFI) mutants were generated by the inverse PCR method using the following primers: *SUMO2* (R59E), 5′-CAGATCGAATTCCGATTTGACGGGCAA-3′ and 5′-ATCGGAATTCGATCTGCCTCATTGACAA-3′; and *SUMO-2* (QFI), 5′-TGGTGGCGGCTAAGGCTAAGAGGCATACACCA-3′ and 5′-CTCTTAGCCTTAGCCGCCACCACAGAACCATCCTG-3′.

### Cell culture and DNA transfection

HeLa cells (RRID: CVCL_0058) were cultured in Ham's F-12 medium (Gibco) containing 10% fetal bovine serum (FBS; Nichirei Bioscience) at 37°C under 5% CO_2_. 293FT cells (RRID: CVCL_6911) were maintained in Dulbecco's modified Eagle's medium (DMEM, high glucose; Gibco) supplemented with 10% FBS (Gibco), 0.1 mM non-essential amino acids (Gibco), 100 U/ml penicillin (Gibco), 1 μg/ml streptomycin (Gibco), 29.2 μg/ml l-glutamine (Gibco) and 100 μg/ml G418 (Promega) at 37°C under 5% CO_2_.

Transfection of plasmid DNA into HeLa cells was performed using the calcium phosphate method for immunofluorescence. Specifically, 10^5^ cells were seeded in 12-well plates and cultured overnight. The following day, DNA/calcium phosphate precipitates containing 3 μg of the indicated expression plasmids were added to each well. After 4 h, the precipitates were removed and cells were cultured for 24 or 36 h in the medium. DNA transfection into 293FT cells was performed using Lipofectamine 2000 (Invitrogen) according to manufacturer's instructions. Basically, 3×10^6^ cells were seeded in 6-well plates and cultured overnight. The following day, a DNA and Lipofectamine mixture containing 4 μg of plasmid DNA and 10 μl of Lipofectamine 2000 were added to cells, which were cultured for the appropriate period.

### Cell synchronization

We used several methods for cell synchronization. To synchronize cells at the G1/S transition, we applied thymidine-hydroxyurea blocks. HeLa cells were pre-synchronized in S phase by incubation with 2.0 mM thymidine (Sigma-Aldrich) for 20 h, released in drug-free medium for 8 h, then cells were resynchronized at the G1/S transition by incubation with 1.5 mM hydroxyurea (Sigma-Aldrich) for 13 h. When needed, plasmid DNA was transfected at 2 h after release from thymidine. The G1/S cells were then washed, incubated in fresh medium for the appropriate time, and used for experiments.

For mitotic time course analysis, cells were arrested at prometaphase using hydroxyurea-monastrol or hydroxyurea-nocodazole blocks. The cells were pre-synchronized at the G1/S transition by incubation with 1.5 mM hydroxyurea for 20 h, released for 3 h and then synchronized at the early phase of mitosis by treatment with medium containing 100 nM monastrol (Sigma-Aldrich), an inhibitor of the mitotic kinesin Eg5 (Kapoor et al., 2000) or 100 ng/ml nocodazole for 6 h. Cells at prometaphase were collected by the shake-off method (Jackman and O'Connor, 2001), aliquoted, and cultured in fresh medium for the indicated period. The cells were then immediately lysed in sample buffer.

### Cytospinning of telophase cells

HeLa cells were arrested at prometaphase by incubation with monastrol for 6 h. Prometaphase cells were then collected by shake-off, seeded in 12-well plates and cultured for 75–85 min, at which point almost all cells had entered telophase as judged by observation under a microscope. Then, cells were trypsinized and collected by centrifugation at 1000 ***g*** for 10 min at 4°C. The cells were suspended in 75 mM KCl at the concentration of 2×10^5^ cells/ml for 5 min, then 0.5 ml of the cell suspension was loaded into a centrifugal cell collector (model SC-2, TOMY Seiko Co., Ltd.). After centrifugation at 800 ***g*** for 3 min, cells attached to the glass slide (Matsunami Glass Ind., Ltd) were fixed with 4% paraformaldehyde (FUJIFILM Wako Pure Chemical Co.) in phosphate-buffered saline (PBS) for 15 min at room temperature and immediately used for immunofluorescence staining.

### Antibodies

The mouse anti-human lamin A/C monoclonal antibody (RRID: AB_627875, 1:2000 and 1:500 dilution for western blotting and immunofluorescence staining, respectively), mouse anti-Myc monoclonal antibody (RRID: AB_2313613, 1:500 dilution), and rabbit anti-cyclin B1 polyclonal antibody (RRID: AB_2072134, 1:5000 dilution) were purchased from Santa Cruz Biotechnology. The rat anti-HA monoclonal antibody (RRID: AB_2314622, 1:500 dilution), mouse anti-FLAG monoclonal antibody (RRID: AB_259529, 1:5000 and 1:500 dilution for western blot and immunofluorescence, respectively), the mouse anti-FLAG antibody (clone M2) labeled with Cy3 (RRID: AB_439700, 1:500 dilution) were obtained from Sigma-Aldrich. The rabbit anti-phospho-lamin A/C (Ser22) monoclonal antibody (RRID: AB_2798221, 1:2000 and 1:2500 dilution for western blot and immunofluorescence, respectively), rabbit anti-SUMO-1 polyclonal antibody (RRID: AB_10698887, 1:500 dilution for western blot) and rabbit anti-SUMO-2/3 monoclonal antibody (clone 18H8) (RRID: AB_2198425, 1:500 dilution for western blot) were purchased from Cell Signaling Technology. The rabbit anti-RepoMan polyclonal antibody (RRID: 17701-1-AP, 1:200 dilution) was purchased from Proteintech. In addition, HRP-conjugated secondary antibodies specific to rat, mouse and rabbit immunoglobulin G (IgG) (RRID: AB_772207, AB_772193 and AB_772206, respectively, 1:7000 dilution) were obtained from GE Healthcare Life Sciences. Anti-rat-IgG, anti-mouse-IgG or anti-rabbit-IgG species-specific antibody conjugated to Alexa Fluor 594 (RRID: AB_10561522, AB_2534087, and AB_2534116), Alexa Fluor 488 (RRID: AB_2534074, AB_2534084, and AB_2534114), and Alexa Fluor 647 (RRID: AB_141778, AB_2535806, and AB_2535814) were purchased from Thermo Fisher Scientific. All Alexa Fluor-conjugated antibodies were used at 1:500 dilution.

### Immunofluorescence and fluorescence microscopy

Cells were grown on glass coverslips, washed with PBS, fixed by incubating in 2% paraformaldehyde (FUJIFILM Wako Pure Chemical Co.) in PBS for 20 min, permeabilized in PBS containing 0.3% Triton X-100 for 15 min, and blocked with 2% bovine serum albumin (BSA; Sigma-Aldrich) in PBS. The coverslips were then incubated overnight with the appropriate primary antibodies diluted in PBS containing 0.1% BSA at 4°C. After five washes with PBS, the coverslips were incubated for 1 h with Alexa Fluor 488-, Alexa Fluor 594- or Cy3-conjugated secondary antibodies in PBS containing 0.1% BSA at 4°C. DNA was stained with Hoechst 33258 dye (Calbiochem). After five washes with PBS, the coverslips were mounted on glass slides spotted with ProLong Gold and SlowFade Gold antifade reagents (Life Technologies). The fluorescence was then visualized by confocal laser scanning immunofluorescence microscopy (TCS SP8, Leica). Multicolor images (1024×1024 pixels) were saved as TIFF files in LAS X Software (Leica) Image processing was performed using Adobe Photoshop software. Fluorescence intensity was measured in the whole cell using Fiji software.

### Immunoprecipitation and western blotting

Cells were washed with PBS and lysed in lysis buffer containing 20 mM HEPES pH 7.4, 150 mM NaCl, 0.5% NP-40, 1 mM DTT, 0.1 mM EDTA, 20 mM N-ethylmaleimide, 1 mM Na_3_VO_4_, 5 mM NaF, 1 mM sodium pyrophosphate, 0.5 mM p-amidino-PMSF and protease inhibitor cocktail (Roche) for 5 min on ice. The lysates were subjected to sonication (SHARP UT-106H) at 4°C and centrifugation at 15,000 ***g*** for 20 min at 4°C. The supernatant was transferred to a new tube and mouse monoclonal anti-HA antibody (clone TANA2, MBL)-magnetic beads or mouse monoclonal anti-FLAG antibody (clone M2, Sigma)-magnetic beads were added at a final concentration of 25 μg/ml. After incubation for 30 min at 4°C with rotation, the magnetic beads conjugated with antibodies were washed with lysis buffer supplemented with 100 mM KCl five times and boiled in Laemmli's sample buffer for 5 min. For immunoprecipitation of endogenous RepoMan, rabbit anti-RepoMan polyclonal antibody was added to the cell lysate at a final concentration of 1 µg/ml. After incubation for 30 min at 4°C, a mixture of protein G- and protein A-Dynabeads (1:1, v/v; Thermo Fisher Scientific) was added to the lysate and rotated for 30 min at 4°C. Dynabeads were washed with lysis buffer supplemented with 100 mM KCl five times and boiled in Laemmli's sample buffer for 5 min. After brief centrifugation (1000 ***g*** for 1 min), the supernatants were subjected to SDS-PAGE and transferred to Immobilon-P membranes (Merck Millipore). Blots were incubated with the indicated primary antibodies in Tris-buffered saline (TBS) containing 2% skim milk and 0.05% Tween 20 and then with appropriate secondary antibodies conjugated with HRP. Bands were detected using an ECL western blotting detection kit (Thermo Fisher Scientific) or a SuperSignal West Femto detection kit (Thermo Fisher Scientific). ECL signals were quantified using ImageJ. Protein concentrations were quantified using a protein assay kit (Bio-Rad).

### Statistical analysis

Statistical significance was determined using unpaired two-tailed Student's *t-*tests. *P*<0.05 was considered statistically significant.

## Supplementary Material

Supplementary information
