# Supplementary material for: SUMOylation of RepoMan during late telophase regulates dephosphorylation of lamin A
Source: J Cell Sci. 2021 Sep 9;134(17):jcs247171. doi: 10.1242/jcs.247171 (PMC8445599; doi:10.1242/jcs.247171)
Supplement: Supplementary information [file joces-134-247171-s1.pdf]

Supplementary information

Table S1 represents conservation of amino acid sequences of a putative SUMO acceptor site in RepoMan polypeptides from human, mouse, rat, cow and dog. Fig. S1 shows that depletion of RepoMan impaired dephosphorylation of mitotic lamin A phosphorylation at Ser392 (related to Fig. 3). Fig. S2 shows HeLa cells after release from monastrol. Cells were attached to the cover glass by cytospinning, fixed and stained with Hoechst 33258 dye.

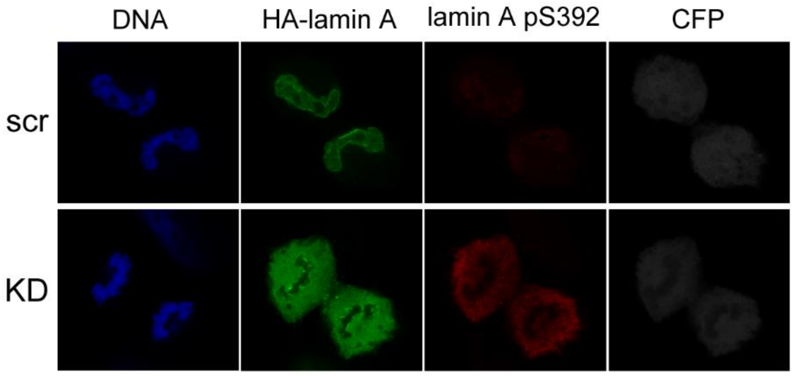

Figure S1. Knockdown of RepoMan delays dephosphorylation of lamin A at pS392. HeLa cells were transfected with shRNA plasmid (scr or KD), HA-lamin A plasmid, and E-CFP plasmid, then synchronized by thymidine-hydroxyurea-nocodazole block. At 90 min after the release, cells were immunofluorescently stained using anti-HA (green) and anti-lamin A/C pS392 (red) antibodies. The images are representative telophase cells examined in two independent experiments (telophase, 6 cells; cytokinesis, 6 cells).

Journal of Cell Science • Supplementary information

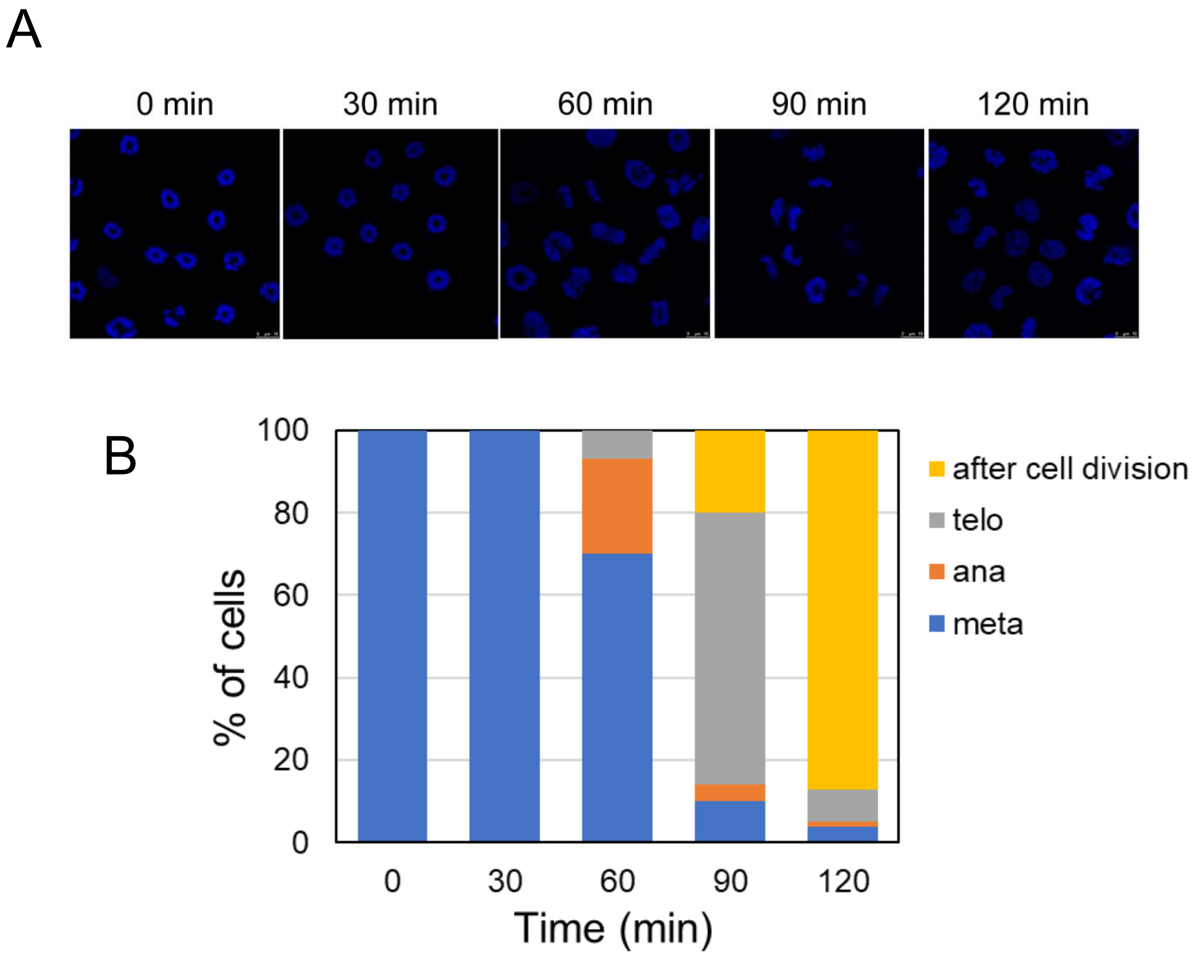

Figure S2. Representative images of cells after release from the hydroxyurea-monastrol blockage. Hela cells were synchronized by the hydroxyurea-monastrol method. Prometaphase cells were collected by shake-off, seeded and cultured in 12-well plates for the indicated time. **(A)** Cells at the indicated time point directly were attached to the cover glass by cytospinning, fixed and stained with Hoechst 33258 dye. **(B)** Histograms represent the cell population at each mitotic stage. n=100 cells per sample, two independent experiments.

Journal of Cell Science • Supplementary information

Table S1. The consensus motif for SUMOylation is well conserved among mammals.

species	amino acid sequences	position of putative SUMO accepetor sites
human	ISPDLNIKCERKDDF	762
mouse	VSESMNLKCEKESEC	741
rat	ISESMNIKCEKESEC	738
cow	ISEDMNIKCEKQSGF	753
dog	ISEDVNIKYEKQDDF	772
